# Membrane-to-Patient Optimization: Individualized Dialyzer Selection for Extracorporeal Dialysis

**DOI:** 10.3390/toxins18040156

**Published:** 2026-03-25

**Authors:** Mariana Murea, Alaa S. Awad, Vandana D. Niyyar, Tibor Fülöp, Akihiro C. Yamashita, Tadashi Tomo, Masanori Abe

**Affiliations:** 1 Department of Internal Medicine, Section on Nephrology, Wake Forest School of Medicine, Winston-Salem, NC 27157, USA; 2 Division of Nephrology, University of Florida, Jacksonville, FL 32209, USA; alaa.awad@jax.ufl.edu; 3 Division of Nephrology, Department of Medicine, Emory University, Atlanta, GA 30322, USA; vniyyar@emory.edu; 4 Division of Nephrology, Department of Medicine, Medical University of South Carolina, Charleston, SC 29425, USA; tiborfulop.nephro@gmail.com; 5 Nephrology Section, Medical Services, Ralph H. Johnson VA Medical Center, Charleston, SC 29401, USA; 6 Department of Chemical Science and Technology, Faculty of Bioscience and Applied Chemistry, Hosei University, Tokyo 184-8584, Japan; yama@hosei.ac.jp; 7 Faculty of Medicine, Clinical engineering Research Center, Oita University, Oita 879-5593, Japan; tomo@oita-u.ac.jp; 8 Division of Nephrology, Hypertension, and Endocrinology, Department of Internal Medicine, School of Medicine, Nihon University, Tokyo 173-8610, Japan

**Keywords:** dialyzer, hemodialysis, hemodiafiltration, inflammation, residual kidney function

## Abstract

Extracorporeal dialysis for uremic toxin removal and fluid regulation relies on specialized dialyzers whose membranes differ markedly in polymer chemistry, pore architecture, adsorption capacity, surface bioactivity, and convective performance. These structural and material distinctions result in wide variation in the clearance of chemically diverse uremic solutes. Despite the expanding range of dialyzer options, membrane selection in clinical practice remains largely non-individualized. In this review, we propose a phenotype-based model for dialyzer membrane selection. We outline how distinct membrane families achieve differential solute clearance and integrate these functional characteristics into a framework that considers residual kidney function, nutritional and inflammatory status, cardiovascular physiology, protein-bound toxin burden, and hemodynamic vulnerability. Because access to advanced membranes varies across regions and dialysis providers, implementation will require adaptation to local formulary constraints. Nevertheless, aligning membrane properties with patient-specific toxin profiles offers a promising strategy to optimize extracorporeal therapy and improve outcomes in chronic dialysis.

## 1. Introduction

The biological complexity of uremic toxicity continues to challenge the efficacy of extracorporeal dialysis. Hemodialysis (HD) and hemodiafiltration (HDF) remain the cornerstone modalities; however, their ability to clear the broad spectrum of retained solutes is fundamentally limited by the dialyzer membrane—the true biochemical interface between patient and treatment [1]. Over the past decade, membrane technology has advanced rapidly, with innovations that differ substantially in pore architecture, sieving characteristics, adsorption capacity, and biocompatibility [2,3,4]. Such properties influence not only solute clearance profiles but also inflammatory responses, oxidative stress, complement activation, and ultimately clinical outcomes.

In this review, we synthesize current knowledge on uremic toxin biology, membrane engineering, solute transport mechanisms, and associated clinical outcomes to propose a phenotype-driven framework for individualized dialyzer selection. While this review focuses on dialyzer membrane characteristics, membrane choice represents only one determinant of dialysis adequacy and individualization. Treatment duration and frequency are other factors that determine solute depuration and should be co-optimized with membrane properties in individualized prescriptions. Because practice patterns and device availability vary globally—conventional high-flux HD remains predominant in many settings, and access to on-line HDF (which requires ultrapure water and certified hemodiafilters) and to adsorptive membranes is region- and provider-dependent—our framework should be viewed as guidance to be adapted to local formulary, reimbursement, and infrastructure constraints.

## 2. Mechanisms of Uremic Toxin Removal in Extracorporeal Dialysis

Over 150 uremic toxins have been identified, spanning diverse physicochemical properties, biological origins, and pathogenic effects [5,6,7,8]. Contemporary classifications consider molecular size, protein-binding affinity, charge, hydrophobicity, origin, and post-translational modifications. Table 1 lists the major uremic retention solutes, their molecular characteristics, protein-binding properties, and primary removal mechanisms.

**Figure 1:**
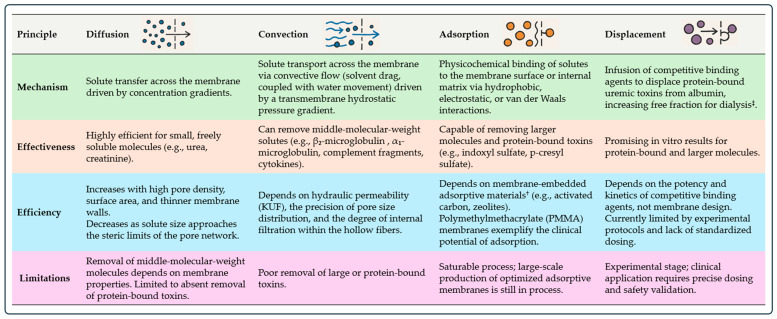
Mechanisms of toxin removal in extracorporeal dialysis. Note: “Solute” refers to any substance dissolved in a solvent (e.g., blood plasma) for removal during dialysis. “Molecule” denotes the chemical entity itself, which may be small (e.g., urea, creatinine), middle-sized (e.g., alpha-1 microglobulin; beta-2 microglobulin), or protein-bound (e.g., indoxyl sulfate, p-cresyl sulfate). The solute can be a molecule (e.g., urea), an ion (e.g., sodium, chloride), or even a macromolecule (e.g., albumin). “Toxins” specifically indicate uremic retention solutes that exert biological harm, including small molecules, middle molecules, and protein-bound uremic toxins. ^†^ Adsorptive membranes (also called mixed matrix membranes [MMMs]) incorporate adsorptive fillers (e.g., activated carbon, zeolites) into polymer matrices. Dual-layer hollow fibers are under experimentation to prevent direct blood contact with adsorbents. ^‡^ Examples of competitive binding agents include tryptophan, loop diuretics, medium-chain fatty acids, salvianolic acids, and ibuprofen.

**Figure 2:**
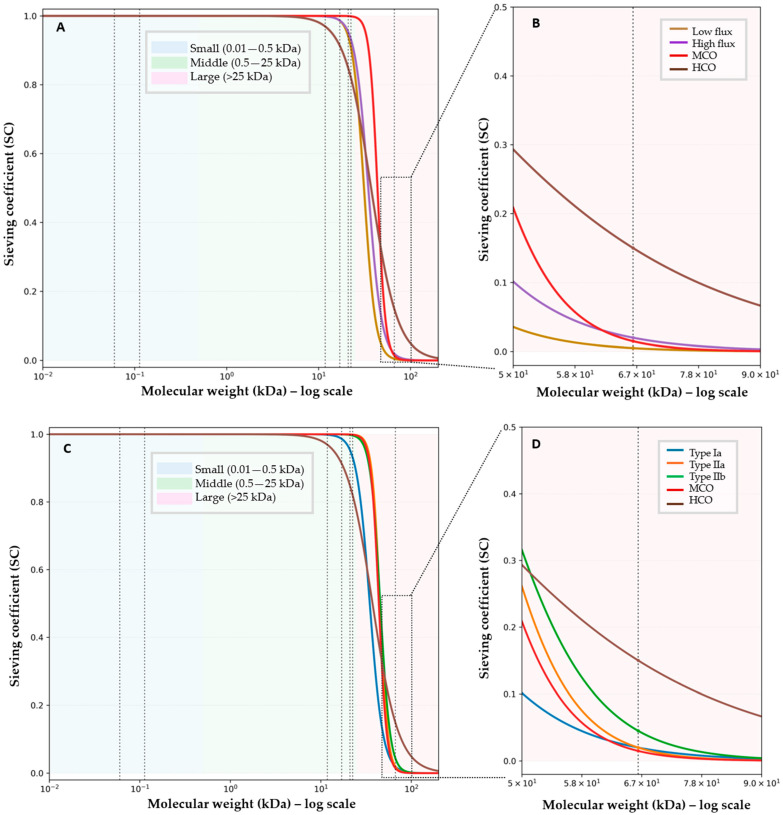
Conceptual sieving curves by dialyzer category with albumin-region zoom. Panel (**A**) displays sieving coefficient (SC) versus molecular weight (kDa, log scale) between international flux classes and cut-off membranes. Panel (**C**) contrasts SC between Japanese classes and cut-off membranes. Background shading colors denote molecular-size ranges. Vertical dashed guides mark representative solutes (from left to right): urea (0.06 kDa), creatinine (0.113 kDa), β_2_-microglobulin (11.8 kDa), TNF-α (~17 kDa), IL-6 (~21 kDa), κ light chain (~22.5 kDa), and albumin (66.5 kDa). The dashed rectangle outlines the magnified region. Panels (**B**,**D**) represent a magnified view of the 0.0–0.5 SC and 50–90 kDa molecular-weight region. The dashed vertical line at 66.5 kDa marks albumin. Notes: Curves are anchored to representative albumin SC values by category; transition steepness reflects pore size distribution (tighter → steeper). Low-flux membranes (dark yellow) demonstrate a sharp decline in SC at relatively low molecular weights, reflecting small pore size, diffusion-dominant transport, and negligible clearance of middle molecules. Albumin SC remains near zero, but middle-molecule permeability is limited. High-flux membranes (purple) exhibit a rightward shift of the sieving curve with increased SC for middle molecules, reflecting larger pores and enhanced convective transport while maintaining low albumin permeability. The transition zone is broader than in low-flux membranes, allowing β_2_-microglobulin clearance with minimal albumin loss. Type Ia (blue) and Type IIa (orange) exhibit low albumin SC; Type IIa shows a steeper cut-off consistent with tight/very narrow pore distribution. Medium-cut-off (MCO) membranes (red) demonstrate a similarly steep cut-off with preserved albumin retention but extend permeability into the large–middle-molecule range (≈25–45 kDa) through optimized internal filtration rather than increased pore heterogeneity. Type IIb (green) has a gentler transition (moderate–wide distribution). High-cut-off (HCO) membranes display a markedly broadened transition zone with elevated albumin SC, reflecting pore sizes that approach the albumin molecular radius and result in clinically significant albumin loss, which restricts their use to acute indications. Type Ib dialyzer is not displayed due to insufficient published characterization to conceptualize a representative sieving curve.

Extracorporeal removal occurs through four mechanisms: diffusion, convection, adsorption, and displacement (Figure 1) [4,5,6,7,8,9,10]. The efficiency and interplay of solute removal are largely determined by dialyzer membrane characteristics, summarized in Table 2 and detailed below. Small water-soluble solutes such as urea and creatinine are readily cleared by diffusion, even with low-flux membranes, due to their low molecular weight and hydrophilicity [9]. Diffusive transport is enhanced by high pore density, large surface area, and thin membranes, but becomes inefficient as solute size approaches pore limits. Middle molecules, in contrast, require larger and uniformly distributed pores for effective clearance. Their accumulation drives inflammation, cardiovascular injury, and dialysis-related amyloidosis, making convective transport essential [10,11]. High-flux and super-high-flux membranes leverage convection, while membranes called ‘medium-cut-off’ (MCO) optimize internal filtration without excessive albumin loss [12]. The boundary between desirable middle-molecule removal and unwanted protein leakage depends on pore size sharpness and uniformity [13]. To visualize how membrane architecture governs solute selectivity, Figure 2 presents conceptual sieving curves comparing different types of dialyzers.

Protein-bound uremic toxins (PBUTs) present unique challenges since only the unbound fraction can cross membranes. Their clearance relies on adsorption, which is influenced by polymer chemistry, surface hydrophilicity, charge distribution, and microdomain architecture [14,15]. Displacement is an emerging concept stimulated by the challenge of protein-bound uremic toxins. Competitive binding agents temporarily displace toxins from albumin, increasing the freely circulating fraction accessible to diffusion or adsorption [11,16,17]. Finally, large–middle molecules, including free light chains, myoglobin, and certain pro-inflammatory mediators, require higher permeability or internal convection to be removed effectively.

## 3. Dialyzer Engineering Principles

Dialyzers have advanced substantially in their material composition, surface chemistry, and structural engineering, all of which influence pore architecture, biocompatibility, and adsorption capacity [18]. As summarized in Table 3, each advancement in membrane engineering has progressively improved the removal of uremic toxins at the higher end of the spectrum—those with larger molecular weight and protein-binding affinity.

Material composition has evolved from early cellulosic designs to advanced synthetic polymers. Unmodified cellulose membranes, such as Cuprophane, were hydrophilic but triggered strong complement activation and provided only diffusive clearance of small solutes [2]. Acetylation in modified cellulose membranes improved biocompatibility but did not overcome limitations in permeability and convective performance compared with synthetic polymers [2,18]. The transition to synthetic membranes marked a major engineering advance. Polysulfone (PS) and polyethersulfone (PES), often blended with hydrophilic additives like polyvinylpyrrolidone (PVP), became dominant due to their mechanical stability, thermal resistance, and ability to support high hydraulic permeability [3]. These membranes underpin high-flux and HDF dialyzers by combining strong diffusive and convective transport with low albumin sieving. Other synthetic polymers, such as polyacrylonitrile (PAN), polyester polymer alloy (PEPA), and ethylene-vinyl alcohol (EVAL), offer favorable hemodynamic compatibility and are used primarily in Japan and Europe [4]. Polymethylmethacrylate (PMMA) membranes occupy a unique niche. Their microporous matrix contains internal adsorptive domains that bind hydrophobic and protein-bound uremic toxins, including cytokines, while maintaining excellent biocompatibility [19]. These properties make PMMA suitable for patients with inflammation, malnutrition, or high protein-bound uremic toxin burden, consistent with its classification as Type S (adsorptive) in the Japanese system [20].

Advances in pore-engineering and fiber-spinning technologies have enabled the development of MCO membranes that feature tightly controlled pore size distributions and enhanced internal filtration to expand clearance to large–middle molecules (25–45 kDa) without clinically significant albumin loss [21,22]. At the upper end of the permeability spectrum, high-cut-off (HCO) membranes demonstrate pore diameters approaching the size of albumin, extending clearance to solutes up to ~60 kDa [23]. Although highly effective for free light chains, myoglobin, and other large inflammatory mediators, their broader pore size distribution results in significant albumin loss, restricting their use to acute indications such as multiple myeloma cast nephropathy and rhabdomyolysis [24].

Hemodiafilters designed for on-line HDF represent a distinct subset of high-performance synthetic membranes engineered to sustain large convection volumes and high transmembrane pressures. Compared with standard HD dialyzers, HDFs typically incorporate thinner fiber walls, larger effective surface areas, and substantially higher hydraulic permeability (KUF), enabling controlled internal filtration and augmented middle-molecule transport [25]. Their pore size distribution must remain sufficiently uniform to prevent clinically significant albumin loss despite high convective fluxes, and due to the direct infusion of replacement fluid, HDF-certified devices undergo additional endotoxin retention testing and biocompatibility validation to meet regulatory requirements. Although many hemodiafilters are constructed from similar base polymers as high-flux dialyzers (e.g., PS or PES), their structural and hydraulic performance characteristics are measurably different, resulting in enhanced clearance of solutes such as β_2_-microglobulin, α_1_-microglobulin, and other middle molecules [25].

## 4. Current Dialyzer Classifications

Dialyzers are classified according to how membrane material and engineering features influence diffusion, convection, adsorption, and albumin retention. The most widely used frameworks include the international system and the Japanese system (Table 4).

The international classification system separates the dialyzers primarily according to flux characteristics [2,10,26,27]. Low-flux dialyzers, traditionally composed of modified cellulosic or early-generation synthetic membranes, feature limited pore size distributions and rely predominantly on diffusion, making them suitable only for small-solute clearance. High-flux dialyzers, which emerged following advances in synthetic polymer engineering, incorporate larger and more uniform pores, higher ultrafiltration coefficients, and improved hydraulic permeability, enabling effective convective removal of middle molecules and supporting modalities such as on-line HDF. Although the international system groups high-flux dialyzers and HDF-compatible hemodiafilters together, only a subset of high-flux membranes meet the engineering and regulatory criteria for on-line HDF. Certified hemodiafilters are designed to deliver convection volumes ≥20–24 L per session, incorporate reinforced hollow fibers, and demonstrate reliable endotoxin retention properties in accordance with on-line HDF safety standards [28]. Despite the simplicity and widespread use of the international classification system, this system does not account for clinically relevant attributes such as adsorption capacity, pore size uniformity, or controlled albumin passage [26].

The original Japanese classification system categorized dialyzers into Types I–V based on β_2_-microglobulin clearance [17]. Type I represented low-flux membranes, while Types II–V indicated progressively higher middle-molecule permeability, with Type V achieving super-high-flux performance (β_2_-microglobulin clearance ≥ 70 mL/min). This approach recognized β_2_-microglobulin as a clinically significant middle molecule linked to inflammation, mortality, and dialysis-related amyloidosis [11,29,30]. By prioritizing β_2_-microglobulin clearance, the Japanese system provided a toxin-oriented framework that better reflected solute removal profiles than the international low-/high-flux dichotomy.

As dialyzer materials, surface chemistry, and pore-engineering advanced, Japan adopted an updated classification system that builds on the original framework by incorporating membrane composition and albumin permeability. The current scheme designates Type Ia (standard-flux), Type Ib (high-flux with mild albumin passage), Types IIa and IIb (super-high-flux with progressively larger pores and internal filtration), and Type S for adsorptive membranes such as PMMA [11]. This approach acknowledges that adsorption and material-dependent interactions with hydrophobic solutes and protein-bound toxins significantly influence performance. For example, Type S membranes exhibit strong binding affinity for protein-bound uremic toxins and inflammatory mediators while preserving albumin, a feature not captured by flux-based criteria [11,29,30].

Neither classification formally includes MCO membranes, yet their design—narrow pore size distribution, internal filtration, and controlled albumin passage—aligns most closely with the Japanese super-high-flux category (Type IIb) [31,32,33]. HCO membranes exceed the permeability range of both systems because their pores approach albumin size, causing significant albumin loss and limiting use to acute conditions such as myeloma cast nephropathy [34,35,36,37]. Hemodiafilters occupy the high-permeability end of the continuum alongside MCO and super-high-flux designs but maintain stricter albumin-retention thresholds than HCO membranes.

Dialyzer class performance differences are summarized in Table 5, which links material properties to permeability, sieving behavior, and solute removal capacity. Variations in KoA, KUF, β_2_-microglobulin clearance, albumin sieving, and pore size uniformity form the structural and functional basis of both international and Japanese classification systems. However, neither framework fully captures the interplay among membrane material, pore architecture, transport mechanisms, and clinical outcomes. As newer designs—such as MCO membranes, advanced adsorptive polymers, and hybrid configurations—emerge, a more integrated, physiology-driven, and toxin-oriented classification may be needed.

## 5. Evidence from Clinical Studies Evaluating Dialyzer Membranes

Clinical studies assessing dialyzer performance have historically centered on comparisons between high-flux and low-flux membranes, yielding mixed or modest results that failed to indicate advantages of dialyzer mechanistic distinctions. The apparent neutrality of many RCTs could, however, be explained by the absence of phenotype-driven membrane selection: most trials did not target patients with middle-molecule or protein-bound toxin-dominant profiles, populations in which membrane differences are most likely to translate into clinical benefit. Viewed through a mechanistic lens, Table 6 summarizes patterns of relationship between membrane type, solute-removal phenotype, and clinical outcomes [11,17,26,27,29,30,38,39,40,41,42,43,44,45,46].

The HEMO trial compared high-flux with low-flux dialyzers in chronic HD and found no overall difference in all-cause mortality, but did report a significant reduction in cardiac mortality and a trend toward benefit in subgroups with longer dialysis vintage or higher β_2_-microglobulin levels [38]. The MPO Study, which randomized patients with serum albumin <4 g/dL to high- versus low-flux membranes, demonstrated a significant survival benefit with high-flux membranes in the low-albumin subgroup, again suggesting that nutritionally vulnerable and inflammatory phenotypes respond to enhanced middle-molecule clearance [39]. These findings were not observed in higher-albumin cohorts, reinforcing the principle that membrane efficacy is phenotype-dependent [26,27,40].

Residual kidney function (RKF) also modulates the impact of membrane selection. High-flux membranes have not demonstrated a survival advantage in patients with substantial RKF. However, in anuric individuals, high-flux membranes are associated with reduced mortality, underscoring the importance of membrane choice in patients with minimal or absent RKF [27,47].

Large-scale registry data from Japan, encompassing over 240,000 patients classified using the Type I–V dialyzer system based on β_2_-microglobulin clearance, demonstrated lower all-cause mortality associated with Type V (super-high-flux) membranes after multivariable adjustment and propensity score matching [11,29]. International cohort analyses similarly report reduced mortality with super-high-flux and protein-leaking membranes [29]. Observational studies further highlighted the potential benefits of adsorptive membranes. PMMA dialyzers have been associated with reduced adjusted mortality rates compared to PS and other synthetic membranes, particularly after accounting for nutritional and inflammatory status [17,42]. These membranes may confer additional cardiovascular protection by attenuating pro-inflammatory mediators such as soluble CD40 ligand (sCD40L), TNF-α, and IL-6 [48].

Beyond survival, patient-reported outcomes provide additional insight into the clinical relevance of membrane selection. High-flux-with-MCO membranes have been linked to improvements in physical functioning, reduced pruritus, and shorter post-dialysis recovery times compared to conventional high-flux dialysis [43,49]. These advanced membranes also exhibit favorable effects on oxidative stress and endothelial function, as demonstrated by metabolomic and proteomic analyses [50]. A comparative study by Maduell and colleagues systematically evaluated four MCO dialyzers (Phylther 17-SD, Vie-18X, Elisio HX19, and Theranova 400) against high-flux HD and post-dilution HDF in 23 patients undergoing six dialysis sessions each [51]. The study demonstrated that differences in efficacy between dialyzers were minimal for small molecules and solutes up to the size of β_2_-microglobulin. However, substantial differences emerged for larger middle molecules: all four MCO dialyzers achieved significantly higher reduction ratios for myoglobin, κ free light chains, prolactin, α_1_-microglobulin, and λ free light chains compared with high-flux HD, while being slightly inferior to post-dilution HDF. Importantly, the four MCO dialyzers demonstrated similar efficacy to each other, with no significant performance differences between brands. Albumin losses were acceptable across all MCO dialyzers (1.5–2.5 g per session), comparable to HDF and substantially higher than high-flux HD (<1 g). A global removal score integrating clearance across molecular-weight ranges confirmed the superiority of HDx over high-flux HD, with efficacy approaching that of post-dilution HDF. These findings establish that MCO-based expanded hemodialysis (HDx) occupies an intermediate position between high-flux HD and HDF for large-middle-molecule clearance, offering a practical alternative for centers without HDF infrastructure while achieving near-equivalent performance for solutes in the 15–45 kDa range.

Hemodynamic instability requires approaches that limit inflammatory surges and improve hemocompatibility. In this context, high-volume hemodiafiltration (HDF) has emerged as a strategy that extends middle-molecule removal while conferring potential survival benefits. The CONVINCE trial, a pragmatic multinational randomized controlled trial of 1360 patients, showed that high-dose HDF (convection volume ≥ 23 L per session) reduces all-cause mortality by 23% compared with conventional high-flux HD (HR 0.77; 95% CI 0.65–0.93) over a median follow-up of 30 months [52]. Subgroup analyses from CONVINCE and a subsequent individual patient data meta-analysis of five randomized trials (>4000 patients) identified patient characteristics associated with differential benefit [53]. The survival benefit appeared most pronounced in patients without preexisting cardiovascular disease or diabetes mellitus, where HDF showed substantial risk reductions. In contrast, among patients with established cardiovascular disease or diabetes, hazard ratios approached unity, suggesting attenuated benefit in these subgroups. Observational and registry studies complement these findings. A large 2019 Japanese cohort study of approximately 10,000 patients reported that pre-dilution on-line HDF was associated with a 17% reduction in all-cause mortality compared with standard HD, with the greatest benefit observed in patients receiving high substitution volumes (~40 L/session) [54]. A recent individual patient data meta-analysis of five RCTs (*n* = 4153 patients) confirmed that post-dilution on-line HDF reduces all-cause mortality (HR 0.84, 95% CI 0.74–0.95) with a dose-dependent relationship between convection volume and survival benefit [53].

Direct comparative data on pre- versus post-dilution HDF remains limited. A recent study by Xu et al. compared mixed-dilution HDF (which combines pre- and post-dilution) with pure pre-dilution and post-dilution modes, finding that mixed-dilution achieved similar small- and middle-molecule clearance to post-dilution while offering improved transmembrane pressure control [55]. The Kidney Health Initiative consensus notes that post-dilution provides the highest solute clearances for the lowest convection volume and is more cost-effective, while pre-dilution requires approximately twice the convection volume to achieve similar solute clearances [28]. Thus, pre-dilution HDF is typically used when desired convection volumes cannot be achieved in post-dilution mode due to unfavorable hemorheologic conditions. Building on these data, a 2026 Japanese expert consensus proposes a national transition toward optimized post-dilution HDF to enhance resource efficiency while maintaining clinical effectiveness [56]. Parallel recommendations from the 2025 ERA EuDial Working Group conclude that HDF appears associated with improved survival when high convection volumes are consistently achieved, even while acknowledging methodological limitations in the existing evidence base [57]. Together, these studies support the integration of high-volume HDF—particularly post-dilution—as an effective strategy for patients with substantial middle-molecule burden, recurrent intradialytic instability, or persistent inflammation. However, the heterogeneity in modality availability, water quality standards, and regional prescribing norms underscores the need to contextualize HDF adoption within local infrastructure constraints.

HCO dialyzers have been studied in multiple myeloma and rhabdomyolysis but remain largely restricted to research protocols or select centers. Trials show that while HCO membranes reduce serum free light chains, they have not consistently improved dialysis independence or survival compared with high-flux dialysis and carry risks such as infection and albumin loss [36,58,59]. In rhabdomyolysis, pilot studies confirm myoglobin removal without clear clinical benefit [60]. Overall, HCO dialyzers are investigational, and larger studies are needed to define their role and safety.

## 6. Albumin Loss with Extracorporeal Dialysis Filters

As illustrated by the albumin-region magnification of the sieving curves (Figure 2), membranes with similar middle-molecule clearance may differ substantially in cut-off sharpness and albumin permeability. Hypoalbuminemia is a recognized mortality risk factor, yet its interpretation is confounded by inflammation and malnutrition, making it difficult to isolate the impact of dialytic albumin loss [61,62]. A better-quality renal dialysis may improve liver synthesis and counteract ongoing albumin losses. The albumin-region zoom in Figure 2 underscores that tight pore size distributions (e.g., Type IIa and MCO) preserve albumin despite enhanced middle-molecule clearance, whereas broader cut-offs (Type IIb and HCO) are associated with progressively greater albumin loss. Evidence linking albumin leakage to outcomes remains inconsistent, and no definitive threshold for safe loss has been established. In trials comparing high- and low-flux membranes, overall mortality was unaffected, although high-flux dialyzers may reduce cardiovascular mortality and improve outcomes in subgroups with low serum albumin, diabetes, or longer dialysis vintage [27]. Albumin loss with these membranes is minimal (<1 g/session) and not associated with harm.

Albumin losses are greater with protein-leaking, adsorptive, and super-high-flux membranes, but long-term safety remains uncertain. Some studies suggest potential benefits—such as improved anemia correction and enhanced toxin removal—yet routine use is unsupported by outcome data, and tolerable albumin loss for chronic therapy is undetermined [61]. Albumin is also a carrier of protein-bound uremic toxins and a filtering loss through the membrane could be one way of removing protein-bound uremic toxins, provided that loss of albumin does not exceed the liver’s synthesis capacity. MCO dialyzers typically cause moderate loss (1–3 g/session), whereas HCO membranes may exceed 20 g/session, a level generally unacceptable for long-term use [63]. Comparative studies between HD with MCO dialyzer (dubbed expanded HD) and HDF showed similar efficacy for toxin clearance, with higher albumin loss in some MCO membranes but no short-term differences in serum albumin or clinical outcomes [64,65].

Although albumin leakage is more pronounced with MCO and HCO membranes than with high-flux dialyzers, meta-analyses indicate no consistent association with increased all-cause mortality, provided nutritional status is maintained and inflammation is controlled [49,61,66]. Comparative studies have shown similar rates of hospitalization and treatment-related complications between patients treated with high-flux, MCO, and PMMA dialyzers [27]. Furthermore, PMMA membranes, through their biocompatible and adsorptive properties, may offer additional protection against inflammation-mediated complications, potentially contributing to improved cardiovascular and overall outcomes [48].

Overall, current evidence supports the safety of advanced membranes when matched to patient phenotype. Albumin loss <2–3 g/session is generally considered safe; higher losses may be acceptable in select cases prioritizing middle-molecule clearance, though long-term safety remains uncertain. Continuous monitoring of nutritional and inflammatory markers is essential. Further research should clarify the clinical impact of albumin loss, especially with emerging membrane technologies.

## 7. Patient-Centered Dialyzer Selection Framework

**Figure 3:**
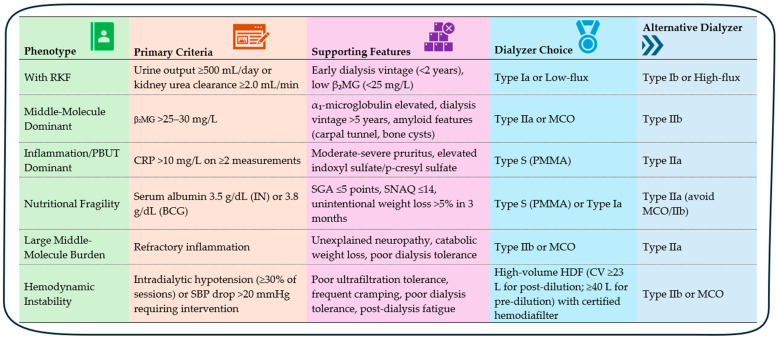
Clinical phenotype-driven dialyzer selection. Notes: In patients with overlapping phenotypes, prioritize dialyzer selection based on the more dominant phenotype. Low-flux dialyzer is acceptable early; reassess as RKF declines or middle-molecule/inflammatory burden emerges. RKF disproportionately clears middle molecules and protein-bound toxins such as indoxyl sulfate and p-cresyl sulfate. Thus, early use of MCO or Type IIb membranes or HDF is generally unwarranted unless baseline inflammatory or amyloidogenic burden is high. CRP thresholds for clinically significant inflammation in hemodialysis patients are not well-standardized; the threshold of CRP > 2× local upper limit of normal on ≥2 measurements (typically >10 mg/L) is proposed to identify patients with inflammation above the typical baseline elevation seen in dialysis populations. Abbreviations: BCG, bromocresol green; CV, convection volume; HCO, high cut-off; HDF, hemodiafiltration; IN, immunonephelometry; MCO, medium cut-off; PBUT, protein-bound uremic toxins; PMMA, polymethylmethacrylate; RKF, residual kidney function; SBP, systolic blood pressure; SGA, Subjective Global Assessment; SNAQ, Simplified Nutritional Appetite Questionnaire; β_2_MG, beta-2 microglobulin.

Uremic toxin accumulation in patients on chronic dialysis varies within and between individuals, reflecting differences in solute physicochemistry, inflammatory activation, nutritional status, RKF, comorbidities, and acute intercurrent illnesses. To align dialyzer membrane properties with biologically anchored phenotypes, we propose a framework for dialyzer selection based on dominant toxin drivers, clinical vulnerabilities, and therapeutic priorities (Figure 3).

Patients with preserved RKF or early dialysis initiation often achieve adequate clearance with standard low- or high-flux membranes (Type Ia/Ib) [11,17,47]. RKF disproportionately clears middle molecules and protein-bound toxins such as indoxyl sulfate and p-cresyl sulfate [27,47,67]. Thus, early use of MCO or Type IIb membranes or HDF is generally unwarranted unless baseline inflammatory or amyloidogenic burden is high [31,49].

Middle-molecule-dominant phenotypes exhibit elevated β_2_-microglobulin, α_1_-microglobulin, osteoprotegerin, complement fragments, and mid-range cytokines, contributing to amyloidosis, vascular stiffness, pruritus, and chronic inflammation—factors linked to mortality and cardiovascular events [17,42]. European Best Practice Guidelines recommend β_2_-microglobulin monitoring and removal [68], and Japanese dialyzer classification is based on β_2_-microglobulin clearance. Patients with β_2_-microglobulin > 25 mg/L or clinical signs of toxin burden may benefit from MCO membranes. Type IIa/IIb designs, with high convective transport and uniform pore architecture, achieve superior clearance of these solutes. MCO membranes extend removal into the 25–45 kDa range via a narrow pore size distribution and optimized hydraulic performance [31,49]. Registry analyses from Japan reporting lower mortality with Type IIa and IIb membranes further support the relevance of enhanced middle-molecule clearance in improving outcomes [29].

Large–middle-molecule phenotypes (30–60 kDa), often marked by high free light chains, IL-6, myoglobin, and fibrinogen fragments, present with refractory inflammation, catabolic weight loss, neuropathy, and poor dialysis tolerance. Type IIb and MCO membranes improve clearance while maintaining acceptable albumin retention [31,49]. HCO membranes provide maximal removal but cause excessive albumin loss, restricting their short-term use in acute conditions such as myeloma cast nephropathy or rhabdomyolysis [36,58,59].

Protein-bound toxin-dominant states involve indoxyl sulfate, p-cresyl sulfate, CMPF, and other hydrophobic solutes that drive cardiovascular risk. Their clearance depends on adsorption rather than permeability. PMMA membranes offer high-capacity binding through a microporous polymer matrix and have demonstrated reductions in inflammatory cytokines and oxidative stress [29]. Surface-modified PS membranes provide moderate adsorption but lack PMMA’s efficacy [11].

Inflammation-dominant phenotypes feature elevated CRP, IL-6, TNF-α, and oxidative stress markers, often with vascular access dysfunction and intradialytic hypotension. Hydrophobic mediators respond to PMMA membranes, while larger cytokines require the convective permeability of Type IIb or MCO membranes. Both have shown biomarker reductions in clinical studies [31,32,33].

Nutritional fragility and hypoalbuminemia demand membranes with minimal albumin leakage. PMMA membranes combine strong adsorption with negligible albumin loss, making them ideal for malnourished patients [48]. PEPA membranes offer an alternative with adsorptive properties and excellent biocompatibility [69], though registry data have not confirmed equivalent mortality benefits in this population [17,42]. Type IIa membranes offer improved middle-molecule clearance with minimal albumin passage, whereas MCO or Type IIb membranes should be reserved for nutritionally stable individuals [27].

Hemodynamic instability requires strategies to mitigate inflammatory surges and improve hemocompatibility. PMMA membranes enhance intradialytic stability, while HDF outperforms high-flux HD in patients with high cardiovascular risk, high infection risk, or inadequate middle-molecule clearance. HDF provides survival and inflammation benefits when high convection volumes (≥23 L/session) are achieved [70,71].

This phenotype-based selection framework is constrained by several evidence gaps. No randomized or observational studies inform dialyzer selection in pregnancy, and physiologic changes in plasma volume, albumin kinetics, and toxin generation preclude extrapolation from nonpregnant populations. Similarly, the safety and efficacy of transitioning patients back to lower permeability membranes after improvement in inflammatory or toxin-dominant states remain unstudied, as the existing literature focuses on outcomes with higher-performance membranes and provides little de-escalation guidance. Published trials also rarely stratify results by evolving nutritional status, RKF, or inflammatory activity, limiting longitudinal decision-making. Finally, operational and resource constraints—such as ultrapure water availability, HDF capability, dialyzer availability, and regional prescribing practices—shape membrane selection. In centers without HDF infrastructure, MCO membranes offer a practical alternative for extended middle-molecule clearance via internal filtration. In centers lacking ultrapure water, equipment, or staffing for HDF, MCO membranes provide a practical alternative, approximating hemodiafilter performance through internal filtration and uniform pore architecture [49,72]. Access to advanced membranes varies globally. Super-high-flux (Type IIa/IIb), PMMA, and certain MCO membranes are widely used in Japan and parts of Europe but are not available in the United States and many low-resource settings. Two advanced dialyzers available in the United States are compared with Japanese dialyzers in Table 7. Fresenius FX CorAL, a next-generation high-flux dialyzer, optimizes middle-molecule clearance while preserving albumin and aligns most closely with the Japanese Type Ib high-flux category. Baxter’s Theranova MCO dialyzer provides clearance of conventional and large–middle molecules up to approximately 45 kDa. Although its functional profile overlaps partially with the large–middle molecule clearance achieved by Japanese Type IIb membranes, Theranova is not a true equivalent, owing to key differences in pore size uniformity and albumin sieving characteristics. Thus, while phenotype-driven selection represents an ideal precision dialysis model, implementation must adapt to local formulary limitations and procurement pathways. Even within these constraints, applying core principles—matching membrane characteristics to toxin burden, inflammatory state, nutritional status, and RKF—can optimize outcomes using available options.

A limitation of this review is its focus on membrane-specific determinants of toxin removal, without detailed consideration of treatment duration and frequency—parameters that independently influence solute clearance and interact with membrane properties. Small water-soluble solutes equilibrate rapidly between compartments and are efficiently cleared even during short sessions, whereas middle molecules and protein-bound toxins require longer treatment times for optimal removal. Protein-bound uremic toxins present a particular challenge because only the unbound fraction crosses dialyzer membranes, and dissociation from carrier proteins is time-dependent [8]. Extended dialysis sessions—whether through nocturnal hemodialysis (6–8 h, 3–6 nights per week) or prolonged intermittent hemodialysis—permit greater cumulative dissociation and enhanced removal. Middle molecules similarly benefit from extended treatment; β_2_-microglobulin clearance increases with session duration due to continued convective transport and equilibration from the interstitial compartment, and high-volume HDF achieves superior middle-molecule clearance in part through sustained convection over longer effective treatment times [1,2,4]. Thus, potential synergies exist between membrane selection and treatment schedule. Patients with protein-bound toxin-dominant phenotypes may benefit from combining adsorptive membranes (e.g., PMMA) with extended session duration, while those with middle-molecule-dominant phenotypes may achieve optimal clearance by pairing MCO or super-high-flux membranes with adequate session length (≥4 h) or high-volume HDF. Conversely, patients with preserved RKF may achieve adequate clearance with standard membranes and conventional schedules, reserving intensified regimens for those with declining RKF or emerging toxin burden. A comprehensive framework integrating membrane selection with individualized treatment duration and frequency remains an important area for future research and guideline development.

## 8. Emerging Membrane Technologies and Strategies

Current dialyzer membranes, despite advances in permeability and biocompatibility, remain limited in their capacity to remove PBUTs, which are strongly associated with cardiovascular morbidity and mortality. Several innovative approaches are under development to address these limitations.

### 8.1. Mixed-Matrix Membranes

Mixed-matrix membranes (MMMs) represent a hybrid technology that incorporates sorbent particles—typically activated carbon—within a polymer membrane matrix [73]. This design combines diffusive and convective transport with adsorptive removal, enabling clearance of PBUTs that conventional membranes cannot effectively eliminate [16]. Preclinical studies have demonstrated that MMMs achieve significantly higher removal of indoxyl sulfate and p-cresyl sulfate compared with conventional high-flux membranes, with one study reporting more than 100% better removal compared to commercial dialysis membranes [73]. Dual-layer MMM designs featuring an inner blood-contacting selective layer and an outer sorbent-containing layer have shown superior removal of protein-bound toxins while maintaining acceptable hemocompatibility [74,75]. In vitro assessments following ISO 10993-4 standards indicate that low-flux MMMs successfully prevent blood contact with activated-carbon particles and demonstrate hemocompatibility comparable to membranes currently used in clinical practice [74]. However, clinical translation remains in early stages, and challenges include optimizing sorbent loading without compromising membrane integrity, ensuring long-term biocompatibility, scaling manufacturing for clinical use, and balancing flux characteristics with albumin retention [75,76].

### 8.2. Electrokinetic and Novel Filtration Approaches

Electrokinetic separation, which exploits differences in solute charge and mobility under an applied electric field, has been proposed for selective toxin removal [73,77]. Early-stage prototypes using electroosmotic flow through charged membranes have demonstrated transport of urea, creatinine, and other uremic solutes, with one study achieving intensive electroosmotic flux of 1–2 mL/cm^2^/h [77]. Ion concentration polarization techniques have shown up to 99.7% retention of albumin while separating neutral metabolites and excess fluid from blood plasma [73]. However, energy requirements, membrane fouling, device complexity, and the need for specialized materials present barriers to clinical adoption. Similarly, biomimetic membranes, nanomaterial-enabled hybrid architectures, and AI-assisted membrane design are being explored but remain largely preclinical [78].

## 9. Conclusions and Future Directions

Clinical studies showed a critical role of membrane characteristics—permeability, adsorptive capacity, and biocompatibility—in shaping outcomes such as mortality, cardiovascular risk, inflammation, and patient-reported quality of life. A phenotype-driven approach to dialyzer selection, informed by RKF, nutritional status, uremic toxin burden, and inflammatory burden, can enable clinicians to optimize solute clearance while minimizing risks such as albumin loss and inflammatory activation. However, membrane selection represents only one dimension of dialysis individualization. Treatment duration and frequency independently influence toxin clearance and may synergize with membrane properties to enhance the removal of middle molecules and protein-bound solutes. Prospective studies evaluating phenotype-driven membrane selection in combination with individualized treatment schedules are needed to establish integrated prescriptions that address the full spectrum of uremic toxicity. Integrating membrane choice into individualized dialysis prescriptions represents a pivotal opportunity to advance therapeutic efficacy, reduce complications, and improve the lived experience of patients with kidney failure. Continued investment in translational research and well-designed clinical trials will be essential to determine whether emerging membrane technologies can meaningfully improve outcomes for patients on chronic dialysis.

## Figures and Tables

**Table 1:** Classification and characteristics of major uremic toxins.

Toxin Category	Representative Solutes	MW (Da)	Protein Binding	Normal Range	Uremic Range (Patients on Chronic Dialysis)	Primary Removal	Pathophysiological Effects
Small Water-Soluble	Urea	60	Minimal	2.5–7.5 mM	15–30 mM	Diffusion	Carbamylation, oxidative stress
	Creatinine	113	Minimal	60–110 μM	800–1200 μM	Diffusion	Marker of kidney function
	ADMA	202	Low	0.4–0.6 μM	1.5–3.0 μM	Diffusion	Endothelial dysfunction, CVD
Middle Molecules	β_2_MG	11,800	Minimal	1–3 mg/L	20–50 mg/L	Convection	Amyloidosis, inflammation, mortality
	α_1_MG	27,000–33,000	Minimal	5–15 mg/L	50–150 mg/L	Convection	Oxidative stress, inflammation
	IL-6	~21,000	Minimal	5 pg/mL	10–50 pg/mL	Convection	Inflammation, CVD
	TNF-α	~17,000	Minimal	10 pg/mL	20–100 pg/mL	Convection	Inflammation, cachexia
Large–Middle Molecules	κ free light chains	~22,500	Minimal	3–19 mg/L	50–500 mg/L	Convection (HCO)	Cast nephropathy, inflammation
	Myoglobin	~17,000	Minimal	70 μg/L	Variable (↑ in rhabdomyolysis)	Convection (HCO)	Acute kidney injury
Protein-Bound	Indoxyl sulfate	213	>90%	0.7–6.3 μM	50–150 μM	Adsorption	CVD, CKD progression, oxidative stress
	p-Cresyl sulfate	188	>95%	0–38 μM	80–250 μM	Adsorption	CVD, mortality, endothelial dysfunction
	CMPF	240	>95%	2 μM	10–30 μM	Adsorption	Endothelial dysfunction

Abbreviations: ADMA, asymmetric dimethylarginine; α_1_MG, alpha-1 microglobulin; β_2_MG, beta-2 microglobulin; CKD, chronic kidney disease; CMPF, 3-carboxy-4-methyl-5-propyl-2-furanpropionic acid; CVD, cardiovascular disease; HCO, high cut-off (dialyzer/membrane category); IL-6, interleukin-6; κ, kappa (light-chain isotype); MW (Da), molecular weight (daltons); TNF-α, tumor necrosis factor-alpha. ↑ denotes elevated.

**Table 2:** Determinants of dialyzer membrane performance.

Characteristic	Description & Role in Performance	Influence on Solute Clearance	Influence on Albumin Retention
Permeability	Ability of membrane to allow passage of water/solutes; depends on pore size, distribution, thickness, and material	Higher permeability increases clearance of small and middle molecules	Excessive permeability risks albumin loss
Pore Size	Diameter of membrane pores; key determinant of molecular selectivity	Larger pores enhance middle molecule clearance	Larger pores increase risk of albumin leakage
Pore Distribution	Uniformity and density of pores across membrane surface	Uniform distribution optimizes clearance	Non-uniformity may cause unpredictable protein loss
Membrane Thickness	Distance solutes must traverse; thinner membranes favor rapid transport	Thinner membranes improve small solute clearance	May increase risk of protein loss if not balanced
Hydraulic Permeability	Capacity for water transport under pressure; measured as ultrafiltration coefficient (KUF)	Higher KUF increases convective clearance	High KUF may increase albumin loss if cut-off is not controlled
Flux	Capacity for convective transport; determined by pore size, distribution, and hydraulic permeability (KUF)	High-flux improves clearance of middle and some large molecules by enabling convection in addition to diffusion	Higher flux increases risk of albumin leakage if molecular-weight cut-off is not tightly controlled
Internal Filtration	Movement of fluid within the dialyzer fibers, as localized filtration and backfiltration without net ultrafiltration ^#^	Boosts middle-molecule clearance during hemodialysis	Risk of albumin loss if pores are large
Molecular-Weight Cut-off	Maximum molecular size allowed to pass; defines selectivity	Higher cut-off allows larger-toxin removal	Must be below albumin molecular weight (~66 kDa) to prevent loss
Sieving Coefficient (SC)	Ratio of solute concentration in ultrafiltrate vs. plasma; reflects membrane selectivity (range: 0–1)	The SC trends high for small solutes, increased for middle molecules with high-flux/HDF/MCO, and low for albumin For convective transport, solute clearance increases with the SC	Plotting of plot SC against the solute size (molecular weight) yields the membrane’s sieving curve A low sieving coefficient for albumin is generally desirable to minimize albumin loss A sharper sieving-curve cut-off (steeper drop in SC with increasing size) is desirable for selective removal of middle molecules while retaining albumin ^§^
Material Composition	Polymer type, hydrophilicity, charge, and surface properties	Affects adsorption, biocompatibility, and selectivity	Influences protein binding and risk of immunologic reactions
Adsorption Capacity	Ability to bind solutes to membrane surface	Enhances removal of protein-bound toxins	May cause non-selective loss of beneficial proteins

Notes: The combination of all characteristic elements determines the balance between effective solute clearance and retention of essential proteins such as albumin. For example, increasing pore size and hydraulic permeability improves middle-molecule removal but risks albumin loss if molecular-weight cut-off and sieving coefficient are not tightly controlled. ^#^ Internal filtration is a passive process within hollow fibers caused by pressure gradients along the dialyzer: at the inlet, higher blood pressure drives fluid from blood to dialysate (filtration); at the outlet, pressure reversal can cause backfiltration from dialysate to blood. This phenomenon is most pronounced in high-flux and MCO membranes with high hydraulic permeability (KUF). It enhances convective clearance of middle molecules. May risk albumin loss. It requires ultrapure dialysate to prevent contamination. Can reach 20–40 mL/min in super-high-flux dialyzers, and is not controlled by dialysis machine. ^§^ Sieving cut-off refers to the transition region on the sieving curve where the SC drops sharply from ~1 (freely passing) to ~0 (effectively retained). A “sharp cut-off” means that the transition is steep (tight selectivity); a “broad/wide cut-off” means the transition is gradual (less selective). Membranes that maintain a very low albumin SC exhibit a sharp cut-off around albumin’s size; higher albumin SC indicates a broader cut-off extending into albumin.

**Table 3:** Dialyzer design parameters.

Membrane Material/Class	Performance Class	Structural Features	Biocompatibility Profile	Solute Transport	Key Design Parameters	Clinical Implications
Cellulosic (unmodified)	Low-flux	Hydroxyl-rich cellulose; hydrophilic	High complement activation	Diffusion only	Low permeability; narrow pore distribution	Obsolete; poor middle-molecule clearance
Modified Cellulose (e.g., cellulose acetate, cellulose triacetate)	Low-flux	Acetylated cellulose; reduced hydroxyl groups	Improved complement profile	Limited convection	Slightly improved permeability	Acceptable biocompatibility; inferior to synthetics
Polysulfone (PS)/Polyethersulfone (PES) + Polyvinylpyrrolidone (PVP)	High-flux	Hydrophobic polymer + hydrophilic additive	Good hemocompatibility; low fouling	Diffusion + convection	High KUF; uniform pores; MW cut-off ~30–35 kDa	Standard high-flux HD; reliable β_2_MG clearance
Polymethylmethacrylate (PMMA)	Adsorptive (Type S)	Microporous adsorptive matrix	Excellent biocompatibility	Adsorption (PBUTs, cytokines)	High adsorption capacity; moderate permeability	Optimal for inflammation, high burden of protein-bound uremic toxins, malnutrition
Polyacrylonitrile (PAN), Polyester polymer alloy (PEPA), Ethylene-vinyl alcohol (EVAL)	High-flux	Synthetic hydrophilic polymers	Good hemodynamic compatibility	Moderate convection; limited adsorption	Moderate permeability; MW cut-off ~30 kDa	Used mainly in Japan/Europe; good middle-molecule clearance
PS/PES	MCO (Medium Cut-Off)	Tight pore size distribution; internal filtration	Good biocompatibility	Convection + large–middle-molecule access	High KUF; internal filtration; MW cut-off ~45 kDa	Alternative to HDF; robust clearance without infrastructure
PS/PES	HCO (High Cut-Off)	Very high permeability (near albumin radius)	Acceptable	Maximal large-molecule removal	Very high KUF; MW cut-off ~60 kDa	Acute use only; albumin loss risk
PS/PES	Hemodiafilters (HF)	Thinner walls; reinforced fibers; endotoxin retention	High biocompatibility	High-volume convection	Very high KUF; uniform pores; certified endotoxin retention	Optimal for HDF; superior β_2_MG, α_1_MG clearance

α_1_MG denotes α_1_-microglobulin; β_2_MG, β_2_-microglobulin; HDF, hemodiafiltration; MW, molecular weight; PBUT, protein-bound uremic toxins.

**Table 4:** Dialyzer classification systems.

Guiding Classification Principle	Category	Defining Criteria	Mechanistic Features	Typical Materials	Clinical Implications
International System					
Permeability- and flux-based classification using KUF and β_2_MG clearance as surrogates for pore size and hydraulic conductance	Low-flux	KUF < 15 mL/h/mmHg; minimal β_2_MG clearance	Diffusion-dominant; minimal convection	Modified cellulose, early PS/PES	Adequate for small solutes; poor middle molecule clearance
	High-flux	KUF ≥ 15; β_2_MG clearance ≥ 15 mL/min	Diffusion + convection; low albumin passage	PS/PES + PVP; PEPA	Enables HDF; improves β_2_MG and middle molecule removal
	Protein-leaking (subset)	Mild albumin leakage	Permeability increases PBUT transport; limited adsorption	Select PS variants	Not widely adopted; niche indications
Original Japanese Classification (Type I–V)					
β_2_MG-clearance-based classification; β_2_MG used as a functional biomarker of middle-molecule permeability and amyloidogenic toxin burden	Type I	β_2_MG clearance < 10 mL/min	Small-solute diffusion only	Cellulose, modified cellulose	Baseline HD
	Type II	β_2_MG clearance 10–20 mL/min	Early middle-molecule permeability	PS/PES	Transitional performance
	Type III	β_2_MG clearance 20–30 mL/min	Improved convection	PS/PES, PEPA	Middle-molecule access increased
	Type IV	β_2_MG clearance 30–50 mL/min	High convection; low albumin loss	High-flux PS/PES	Modern high-flux HD
	Type V	β_2_MG clearance ≥ 70 mL/min	Super-high-flux performance; internal filtration	Advanced PS/PES	Associated with improved outcomes in Japanese registry
Current Japanese Classification (Ia–Ib–IIa–IIb–S)					
Hybrid classification integrating membrane flux, β_2_MG clearance, albumin sieving coefficient, and membrane material (adsorptive vs. synthetic)	Type Ia	Low-flux or lower-performance high-flux dialyzers	Diffusion of small solutes	Modified cellulose, PS	Baseline HD
	Type Ib	High-flux with mild albumin permeability	Moderate convection	PS/PES	Intermediate clearance
	Type IIa	Super-high flux, minimal albumin leakage	Strong convection; uniform pores	High-performance PS/PES	Improved β_2_MG and middle-molecule clearance, similar to MCO performance
	Type IIb	Super-high flux with controlled albumin loss	Access to large–middle molecules	Advanced PS/PES	Improved large–middle-molecule clearance and mild albumin leakage
	Type S	Adsorptive membranes	High PBUT and cytokine adsorption	PMMA	Ideal in inflammation, high protein-bound uremic toxin burden, malnutrition
Hemodiafilters (HF)	Certified for on-line HDF	High KUF; endotoxin retention	High-volume convection	High-performance PS/PES	Strong middle molecule and large–middle-molecule clearance; optimal when HDF feasible
Emerging/Unclassified					
Mechanistic selectivity and pore-architecture-based classification emphasizing internal filtration and albumin-sieving precision	MCO	Uniform pore size distribution; internal filtration	Convection + internal solute drag	Specialized PS/PES	HDx therapy; alternative to HDF; minimal albumin loss
	HCO	Very high permeability; albumin threshold exceeded	Maximal large-middle-molecule clearance	Engineered PS/PES	High albumin loss; restricted to acute indications

β_2_MG denotes β_2_-microglobulin; HD, hemodialysis; HDF, hemodiafiltration; HDx, expanded HD; HCO, high cut-off; KUF, ultrafiltration coefficient; MCO, medium cut-off; PBUT, protein-bound uremic toxins; PEPA, polyester polymer alloy; PES, polyethersulfone; PMMA, polymethylmethacrylate; PS, polysulfone; PVP, polyvinylpyrrolidone.

**Table 5:** Dialyzer functional characteristics by dialyzer category *.

System	Category	KoA (Urea) (mL/min)	KUF	β_2_MG Clearance (mL/min)	Albumin SC	β_2_MG SC	Mean Pore Radius (nm)	Pore Size Distribution ^†^	Dialyzer Names & Manufacturers ^‡^
International	Low (or standard)-flux	500–800	<15	<15	<0.01	<0.2	~3–5	Narrow	FX 8 (Fresenius), Polyflux 14L (Vantive), Elisio-13H (Nipro)
	High-flux	800–1200	20–60	40–60 (HD), up to 80–100 (HDF)	0.01–0.03	0.5–0.8	~5–10	Narrow–Moderate	FX CorDiax 80 (Fresenius), Polyflux 170H (Vantive), Elisio-19H (Nipro), Revaclear 400 (Vantive)
	Protein leaking/adsorptive	900–1200	30–80	60–90	0.05–0.1	0.8–1.0	~10–20	Uniform (adsorptive)	BG-U (Toray, PMMA), Filtryzer BK (Toray, PMMA), Nephral ST (Vantive, AN69-ST)
Japanese 2005	Type I	500–800	<15	<10	<0.01	<0.2	~3–5	Narrow	FX 8 (Fresenius), Polyflux 14L (Vantive) Elisio-15L (Nipro), Sureflux-15L(Nipro)
	Type II	800–1000	15–30	<30	0.01–0.03	0.5–0.7	~5–10	Moderate	Polyflux 17L (Vantive), Sureflux-15G (Nipro), Sureflux-15E (Nipro)
	Type III	1000–1200	30–60	<50	0.02–0.05	0.7–0.8	~10–15	Moderate	Polyflux 170H (Vantive) Elisio-15K (Nipro), CELLENTIA (Nipro),FB-15U (Nipro),
	Type IV	1100–1300	60–80	<70	0.05–0.1	0.8–1.0	~15–20	Moderate–Wide	Revaclear 400 (Vantive), FX-80 (Fresenius), Nephral ST (Vantive, AN69-ST), Polyflux 170H (Vantive), Elisio-21H (Nipro), Sureflux-15UX (Nipro)
	Type V	1200–1300	>80	≥70	0.08–0.15	0.9–1.0	~20–25	Moderate–Wide	Elisio-21HX (Nipro) APS-21EX (Asahi)
Japanese 2023	Type Ia	500–1300	<80	<70	< 0.03	0.5–1.0	~5–20	Narrow–Moderate	FX 8 (Fresenius), Polyflux 14L (Vantive), Elisio-15L (Nipro), Sureflux-15L (Nipro), Polyflux 17L (Vantive), Sureflux-15G (Nipro), Sureflux-15E (Nipro), Polyflux 170H (Vantive), Elisio-15K (Nipro), CELLENTIA (Nipro), FB-15U (Nipro), Filtryzer BK-2.1U (Toray, PMMA), Revaclear 400 (Vantive), FX-80 (Fresenius), Nephral ST (Vantive, AN69-ST), Polyflux 170H (Vantive), Elisio-21H (Nipro), Sureflux-15UX (Nipro)
	Type IIa	1200–1300	>80	≥70	~0.02	0.9–1.0	~20–25	Tight/Very narrow	FX 180 CorDiax (Fresenius), Elisio-21HX (Nipro)
	Type IIb	1200–1300	>80	≥70	~0.04–0.05	0.9–1.0	~20–25	Moderate–Wide	PES-17Dα(Nipro)
	Type S	900–1200	30–80	60–90	0.05–0.1	0.8–1.0	~10–20	Uniform (adsorptive)	BG-U (Toray, PMMA), Filtryzer BK (Toray, PMMA)
Other	Hemodiafilter (HDF)	1100–1500	≥60–100	60–100 (HD), 80–120 (HDF)	0.01–0.03	0.8–1.0	~10–15	Narrow–Moderate	FX CorDiax 100/120 (Fresenius), Polyflux 210H/170H (Vantive), Elisio-19H (Nipro)
	MCO (Medium Cut-Off)	900–1200	35–60	70–100	0.01–0.02	0.9–1.0	~8–15	Tight/Very narrow	Theranova 400/500/600 (Vantive), MCO series (B. Braun)
	HCO (High Cut-Off)	1200–1500	60–120	90–120	0.10–0.20	≈1.0	~20–30	Very wide	HCO 1100/2100 (Baxter/Gambro), SepteX HCO series (Fresenius)

Notes: * Functional characteristics values listed in this table are representative and may vary by specific dialyzer model, manufacturer, and prescription (HD vs. HDF). For precise values, consult manufacturer datasheets and published comparative studies. ^†^ Pore size distribution describes how tightly or broadly pore diameters are spread in the membrane: a narrow (tight) distribution produces a sharper sieving cut-off, while a wide distribution yields a gentler cut-off and greater likelihood of large-pore passage. Tight (very narrow) denotes highly uniform pores, sharp sieving cut-off, typical of modern PES/high-flux and MCO membranes. Moderate distribution denotes broader spread than tight, typical of many conventional high-flux designs. Wide/Very wide distribution denotes broader tail toward large pores, less sharp cut-off, used here for HCO (albumin-permeable). Uniform (adsorptive) indicates homogeneous pore structure with significant adsorption-mediated removal (e.g., PMMA/Type S), not large-pore-driven passage. ^‡^ Examples are based on typical dialyzer models and manufacturers that fit the described classification criteria in the provided table. Actual product availability and classification may vary by region and over time. Contemporary hollow-fiber dialyzers are engineered with a membrane wall thickness of approximately 35 μm and an inner lumen diameter of roughly 200 μm—dimensions optimized to balance hydraulic permeability, mechanical stability, and internal filtration dynamics across membrane types. Exact specifications vary by dialyzer model and manufacturer; detailed performance parameters should be verified from product datasheets. β_2_MG clearance, β_2_-microglobulin clearance, is a marker for middle-molecule removal. KoA (urea) denotes mass transfer area coefficient for urea, indicating dialyzer efficiency for small solutes (mL/min). KUF, ultrafiltration coefficient, represents the parameter of a dialyzer membrane that quantifies its ability to allow water (plasma water) to pass through under a given transmembrane pressure (mL/h/mmHg). The sieving coefficient (SC) can vary between manufacturers and are highly dependent on test conditions (blood flow, ultrafiltration rate, plasma source). Albumin SC values: <0.01 indicates negligible albumin loss; 0.01–0.03 is low; 0.05–0.15 is moderate to high and may result in clinically significant albumin loss. β_2_MG SC values: Higher values indicate better middle-molecule removal. HD, hemodialysis; HDF, hemodiafiltration.

**Table 6:** Summary of randomized controlled trials and large-scale studies comparing dialyzer membrane types and clinical outcomes.

Trial Name [Ref.]	Population Studied	Dialyzers Studied (n/Group)	Follow-Up Duration	Primary Outcome & Results
HEMO Study [38]	Maintenance HD patients (*n* = 1846), US, prevalent	High-flux (*n* = 902) vs. Low-flux (*n* = 944)	Median 2.8 years	All-cause mortality: No significant difference (HR 0.94, 95% CI 0.81–1.10, *p* = 0.44); Cardiac mortality lower in high-flux (*p* = 0.03)
MPO Study [39]	Incident HD patients (*n* = 738), Europe, stratified by albumin	High-flux (*n* = 367) vs. Low-flux (*n* = 371)	Median 3.7 years	All-cause mortality: No difference overall; Lower mortality in high-flux for albumin ≤ 4 g/dL (RR 0.49, 95% CI 0.28–0.87, *p* = 0.032)
EGE Study [41]	Maintenance HD patients (*n* = 704), Europe	High-flux vs. Low-flux	Up to 7.5 years	Fatal/nonfatal CV events: No significant difference (HR 0.73, 95% CI 0.49–1.08, *p* = 0.1)
Japanese Registry (2010–2013) [29]	Maintenance HD patients (*n* = 238,321), Japan	Low-flux, High-flux, Protein-leaking (Type I–V)	3 years	All-cause mortality: Lower in protein-leaking vs. high-flux (HR 0.95, 95% CI 0.92–0.98, *p* = 0.006); Type V lower than Type IV (HR 0.91, 95% CI 0.88–0.94, *p* < 0.0001)
Japanese Registry (2008–2009) [30]	Maintenance HD patients (*n* = 203,008), Japan	Type I–V dialyzers	1 year	All-cause mortality: Type V lower than Type IV (HR 0.93, 95% CI 0.89–0.97, *p* < 0.001)
Japanese Registry (2017–2019) [11]	Maintenance HD patients (*n* = 181,804), Japan	Type Ia, IIa, IIb, S (PMMA)	2 years	All-cause mortality: Lower in IIa (HR 0.91, *p* < 0.001), IIb (HR 0.85, *p* < 0.001), S (HR 0.93, *p* < 0.001) vs. Ia
Japanese Registry (2008–2009) [17]	Maintenance HD patients (*n* = 142,412), Japan	PS, CTA, PES, PEPA, PMMA, PAN, EVAL	1 year	All-cause mortality: Lower in PMMA (HR 0.84, 95% CI 0.76–0.93, *p* < 0.001) and PES (0.88, 95%CI 0.82–0.94, *p* < 0.001) vs. PS after adjustment
Cochrane Meta-analysis [26]	ESKD patients (*n* = 3820), 33 RCTs	High-flux vs. Low-flux	2 years (median)	CV mortality lower in high-flux (RR 0.83, 95% CI 0.70–0.99, *p* = 0.04); no difference in all-cause mortality (RR 0.95, 95% CI 0.87–1.04, *p* = 0.26)
Lim et al. 2020 RCT [43]	Maintenance HD patients (*n* = 49), Korea	MCO (Theranova 400) vs. High-flux (FX CorDiax)	12 weeks	QOL: Physical functioning higher in MCO (*p* = 0.042); pruritus lower in MCO (*p* = 0.034)
Kim et al. 2021 Prospective Study [44]	Maintenance HD patients (*n* = 20), Korea	MCO vs. High-flux (cross-over)	15 weeks	Metabolomics/proteomics: Significant differences in profiles (*p* < 0.05 for key metabolites/proteins)
Cho et al. 2019 Cohort [45]	Maintenance HD patients (*n* = 57), Korea	MCO vs. High-flux	12 months	Middle-molecule reduction ratio higher in MCO; no significant long-term change in serum levels (*p* > 0.05)
Mitchell et al. 2023 Systematic Review [46]	ESKD patients, RCTs and observational studies	HDx (MCO) vs. High-flux HD vs. HDF	12 weeks–1 year (HDx); up to 7 years (HDF)	Mortality: No difference HDx vs. HDF; QoL benefits with HDx in some studies (*p* < 0.05)

**Table 7:** Functional correspondence between U.S.-available dialyzers and the current Japanese classification.

Japanese Category	Closest U.S. FDA-Approved Dialyzer or Category
Type Ia	U.S. low-flux dialyzers (various FDA-cleared models)
Type Ib	Fresenius FX CorAL (high-flux)
Type IIa	No U.S. equivalent available
Type IIb	Baxter Theranova (MCO) is the closest functional neighbor, has partial functional overlap, but is not equivalent
Type S (PMMA adsorptive)	None (PMMA not marketed or FDA-cleared in the U.S.)

## Data Availability

No new data were created or analyzed in this study.

## Abbreviations

The following abbreviations are used in this manuscript:

ADMA	Asymmetric dimethylarginine
α_1_MG	Alpha-1 Microglobulin
β_2_MG	Beta-2 Microglobulin
CMPF	3-Carboxy-4-Methyl-5-Propyl-2-Furanpropionic Acid
CRP	C-Reactive Protein
EVAL	Ethylene–Vinyl Alcohol
HCO	High Cut-Off
HD	Hemodialysis
HDF	Hemodiafiltration
HF	Hemodiafilter
IL-6	Interleukin-6
KoA	Mass Transfer Area Coefficient
KUF	Ultrafiltration Coefficient
MCO	Medium Cut-Off
PAN	Polyacrylonitrile
PBUT	Protein-Bound Uremic Toxins
PEPA	Polyester Polymer Alloy
PES	Polyethersulfone
PMMA	Polymethylmethacrylate
PS	Polysulfone
PVP	Polyvinylpyrrolidone
RCT	Randomized Controlled Trial
RKF	Residual Kidney Function
sCD40L	Soluble CD40 Ligand
TNF-α	Tumor Necrosis Factor Alpha
